# An evaluation of psychometric properties and utility of the Hope Index (HI): a systematic review and meta-analysis protocol

**DOI:** 10.1186/s13643-026-03128-7

**Published:** 2026-03-03

**Authors:** Jermaine M. Dambi, Beatrice K. Shava, Thandiwe Mashunye, Lloyd Dzapasi, Mutsa Mazarura, Dixon Chibanda

**Affiliations:** 1 Friendship Bench Zimbabwe, 4 Weale Road, Milton Park, Harare, Zimbabwe; 2 Rehabilitation Sciences Unit, Faculty of Medicine and Health Sciences, University of Zimbabwe, Avondale, Harare Zimbabwe

**Keywords:** Hope index, Psychometrics, Systematic review, Meta-analysis, Protocol

## Abstract

**Background:**

The number of positive psychological interventions, including hope interventions to foster well-being and decrease the huge burden of mental conditions, has exponentially increased. Consequently, the number of hope outcome measures has also increased. The Hope Index (HI) is an extensively used hope outcome measure, but the evidence of its psychometric performance is fragmented. This systematic review and meta-analysis aims to critically appraise the psychometric properties of the Hope Index and assess its utility.

**Methods/design:**

Two independent and blinded reviewers will search articles in PubMed, Scopus, Web of Science, CINAHL, PsychINFO, ProQuest Dissertations, and Google Scholar. Thereafter, three independent reviewers will then screen the retrieved articles. The risk of bias across studies will then be evaluated using the COnsensus-based Standards for the selection of health Measurement INstruments (COSMIN) checklist. The quality of the psychometric properties will be rated using a pre-set criterion and, after that, synthesized using the modified Grading of Recommendations Assessment, Development, and Evaluation (GRADE) checklist.

**Discussion:**

The proposed systematic review will appraise the collective evidence of the psychometric robustness of the HI. The review will also assist in identifying psychometrics that may require further evaluation and make recommendations on the feasibility and utility of the HI.

**Systematic review registration:**

PROSPERO CRD42024511130.

**Supplementary Information:**

The online version contains supplementary material available at 10.1186/s13643-026-03128-7.

## Background

Mental disorders account for the greatest burden of disability and socioeconomic costs, i.e. direct and indirect costs. For example, per 2019 global burden disease survey, mental disorders accounted 16% of the global disability-adjusted life years (DALYs); this translates to 418 million DALYs [1]. With the ever-increasing burden of mental disorders, there has been a greater call for evidence-based interventions and a call to action for greater investment and prioritisation of mental health. Mental healthcare covers a broad spectrum of interventions, ranging from pharmacotherapy, psychotherapies, complementary treatments (e.g. activity based), and combination therapies (e.g. pharmacotherapy and psychotherapy). Mental healthcare interventions can also be classified as classical (e.g. cognitive behavioural therapies) and positive psychological interventions. The past few decades have seen an exponential increase in positive psychological interventions [2, 3]. Positive psychology interventions broadly aim to maximise human potential and capabilities to optimise functioning and well-being [4, 5]. Positive psychology interventions (PPI) foster flourishing in both individuals and communities and are anchored in strengthening constructs such as resilience, self-efficacy, flourishing, and hope [3, 5]. For instance, per cognitive theories, mental conditions such as depression are resultant of repetitive, negative thoughts and emotions [5, 6]. Within the paradigm of positive psychology, the vicious cycle of negative thoughts and feelings can be counteracted through an infusion of positive feelings and hopeful thinking [3, 6]. As the field of positive psychology continues to evolve, there is accumulative evidence of the impacts of certain constructs (e.g. hope, resilience, gratitude, and character strengths) in optimising mental health [7]. For instance, a meta analysis shows the buffering effects of hope on depressive symptoms with a big effect size: *r* = − 0.391, 95% CI [− 0.429, − 0.351], SE = 0.008, and *p* = 0.001 [3].

Although not universally defined and conceptualised, hope can be defined as a strong desire that is actively pursued, while it is uncertain that this desire will be fulfilled [8]. With the increasing number of promising hope-enhancement interventions, there has been an upsurge in hope outcome measures [3, 9]. Consequently, it is vital to distil the evidence of the psychometric robustness of existing hope outcome measures. An ideal hope outcome measure should be valid and reliable, sensitive to change, feasible in terms of respondent burden (few items with robust content validity), and cheap (ideally available at no to low cost) [10]. The Hope Index (HI) is an extensively applied hope outcome measure [9, 11, 12]. The HI is a 12-item question measuring hope in three domains, i.e. temporality and future (the individual’s belief in the future and their ability to set and achieve goals), positive readiness and expectancy (the individual’s sense of optimism and their belief in their ability to overcome challenges), and interconnectedness (the individual’s sense of connection to others and their belief in the power of relationships) [13]. Scores are rated on a 4-point Likert scale, with qualifiers ranging from 1 (strongly disagree) to 4 (strongly agree) [14]. There are no standard cut-off scores for the HI; higher scores denote high hope evaluations [13]. Originally developed for American adults with cancer, the HI has been extensively used in different populations, including children [15, 16], the elderly [11, 17, 18], the general population [19–21], and populations with specific conditions (e.g. HIV [22, 23], diabetes [24, 25], amongst others).

There have been previous attempts to appraise the psychometric properties of hope scales, including the HI. In their systematic review, Redlich-Amirav et al. identified 18 hope outcome measures across 68 studies, with the Snyder Hope Scale and Hope Index (HI) cited as the most used hope outcome measures [12]. Redlich-Amirav et al. applied a robust methodological approach, particularly article searches, screening, and data extraction. However, the review only evaluated the risk of bias (RoB) across studies but did not evaluate the quality of the psychometrics reported nor collated the evidence of outcomes across studies, thus limiting the study’s external validity [12]. Per best practice, psychometric evaluation reviews should first assess the risk of bias per (RoB) per study per outcome, rate the quality of the psychometric property evaluated, and thereafter synthesise the evidence per psychometric property per outcome using an overarching framework, like the Grading of Recommendations, Assessment, Development, and Evaluations (GRADE) approach [26–28]. Also, another systematic review by Nayeri et al. (2020) explored the construct validity of the HI by qualitatively analysing 13 studies [9]. Although the review provides essential insights into the HI by exploring the outcome measures’ structural validity, construct validity, and reliability, the review’s methodological limitations negatively affect its internal and external validity. First, the review primarily included studies assessing the HI factorial structure. Although structural validity is an essential psychometric property [28], the review by Nayeri et al. misses the opportunity to holistically appraise HI’s psychometric robustness as other psychometric properties (e.g. responsiveness, item invariance) were not evaluated. Second, integrating RoB assessments was not well articulated, as Nayeri et al.’s systematic review provides a generic overall assessment of study quality. Ideally, to negate reporting bias, psychometric evaluation studies must stratify studies according to the degree of RoB, and conclusions should follow study quality [28]. Importantly, Nayeri et al. did not rate the certainty of evidence of the appraised psychometric properties across studies. Ideally, authors should use an overarching frame like the GRADE approach to increase transparency regarding overall evidence synthesis, as it inherently considers RoB and the quality of psychometrics ratings [26–28]. Building on existing work, we propose an updated systematic review and meta-analysis to critically appraise the psychometric performance of the Hope Index and assess its utility. The HI is an extensively applied hope outcome measure, with over 750 studies published between the publishing of the last systematic review. Also, there has been an upsurge in item response theory-based studies evaluating HI performance [21, 29]. However, evidence of psychometric robustness is still fragmented. Whereas previous reviews used narrative analytical approaches, we will use a meta-analytical approach, which will provide more robust evidence of the HI’s validity and reliability, including evidence of the tool’s feasibility and utility across different contexts. In summary, this review seeks to answer the question: in adults across all geographical settings, what is the collective evidence of validity, reliability, and feasibility of the Hope Index?

## Methods

### Protocol registration

We will perform this systematic review and meta-analysis per updated Preferred Reporting Items of Systematic reviews and Meta-Analyses (PRISMA) guidelines [30]. This protocol is reported per PRISMA protocol (PRISMA-P) guidelines [30, 31]. The protocol is registered on the PROSPERO database — ref.: CRD42024511130.

### Eligibility criteria

Table 1 outlines the study inclusion and exclusion criteria. Essentially, we will analyze all quantitative studies using the HI regardless of setting or population group. Formally peer-reviewed articles (e.g. journal articles, postgraduate theses) and pre-prints will be included in the review. We will exclude conference abstracts, reviews, grey literature, and studies published in any other language than English due to translation budgetary restraints.

**Table 1:** Eligibility criteria

Element	Inclusion	Exclusion
Population	Any adult population defined as any persons aged ≥ 18 years	Studies in which adults constitute < 50% of the study sample
Intervention	Any	N/A
Comparison	Any	N/A
Outcome	Evaluating any of the psychometrics per COSMIN taxonomy	
Setting	Any	N/A
Study design	Any quantitative design (e.g., cross-sectional, longitudinal, experimental designs)	
Information sources	▪ Formally peer-reviewed articles (e.g. journal articles, postgraduate theses ▪ Pre-prints	▪ Conference abstracts ▪ Reviews (e.g. systematic and scoping reviews) as we primary want to analyse primary studies and perform a meta-analysis as appropriate ▪ Grey literature
Language	English	Non-English languages (e.g. Spanish, French)
Time	1992 up to penultimate search date	

### Information sources

We will search PubMed, CINAHL, Scopus, PsychINFO, Africa-Wide Information, and other relevant databases for peer-reviewed articles. We will apply a twofold, hierarchical search process. First, databases will be searched individually. Afterwards, using the EBSCO host search engine, we will conduct a combined database(s) search to increase the search strategy sensitivity and specificity. Peer-reviewed databases will be searched from 1992 (the year in which the HI was first published) to July 2024. Additionally, Google Scholar, ProQuest Dissertations, Research Square, and OpenGrey will also be searched for pre-prints and dissertations. Last, we will perform backwards and forward citation searches to increase our search robustness and reach.

### Search strategy

In Table 2, we outline an example pilot search strategy for PubMed. Articles will be searched using Boolean logic operators (1 AND 2) AND (4 NOT 5). The final search strategies across databases will be piloted and finalised in consultation with a subject specialist librarian.

**Table 2:** Draft PubMed search strategy

Search no. #	Key term(s)/constructs	Boolean logic operator
1	Herth Hope Index	1
2	Hope Index	2
3		1 AND 2
4	(instrumentation[sh] OR methods[sh] OR "Validation Studies"[pt] OR "Comparative Study"[pt] OR "psychometrics"[MeSH] OR psychometr*[tiab] OR clinimetr*[tw] OR clinometr*[tw] OR "outcome assessment (health care)"[MeSH] OR "outcome assessment"[tiab] OR "outcome measure*"[tw] OR "observer variation"[MeSH] OR "observer variation"[tiab] OR "Health Status Indicators"[Mesh] OR "reproducibility of results"[MeSH] OR reproducib*[tiab] OR "discriminant analysis"[MeSH] OR reliab*[tiab] OR unreliab*[tiab] OR valid*[tiab] OR "coefficient of variation"[tiab] OR coefficient[tiab] OR homogeneity[tiab] OR homogeneous[tiab] OR "internal consistency"[tiab] OR (cronbach*[tiab] AND (alpha[tiab] OR alphas[tiab])) OR (item[tiab] AND (correlation*[tiab] OR selection*[tiab] OR reduction*[tiab])) OR agreement[tw] OR precision[tw] OR imprecision[tw] OR "precise values"[tw] OR test–retest[tiab] OR (test[tiab] AND retest[tiab]) OR (reliab*[tiab] AND (test[tiab] OR retest[tiab])) OR stability[tiab] OR interrater[tiab] OR inter-rater[tiab] OR intrarater[tiab] OR intra-rater[tiab] OR intertester[tiab] OR inter-tester[tiab] OR intratester[tiab] OR intra-tester[tiab] OR interobserver[tiab] OR inter-observer[tiab] OR intraobserver[tiab] OR intra-observer[tiab] OR intertechnician[tiab] OR inter-technician[tiab] OR intratechnician[tiab] OR intra-technician[tiab] OR interexaminer[tiab] OR inter-examiner[tiab] OR intraexaminer[tiab] OR intra-examiner[tiab] OR interassay[tiab] OR inter-assay[tiab] OR intraassay[tiab] OR intra-assay[tiab] OR interindividual[tiab] OR inter-individual[tiab] OR intraindividual[tiab] OR intra-individual[tiab] OR interparticipant[tiab] OR inter-participant[tiab] OR intraparticipant[tiab] OR intra-participant[tiab] OR kappa[tiab] OR kappa's[tiab] OR kappas[tiab] OR repeatab*[tw] OR ((replicab*[tw] OR repeated[tw]) AND (measure[tw] OR measures[tw] OR findings[tw] OR result[tw] OR results[tw] OR test[tw] OR tests[tw])) OR generaliza*[tiab] OR generalisa*[tiab] OR concordance[tiab] OR (intraclass[tiab] AND correlation*[tiab]) OR discriminative[tiab] OR "known group"[tiab] OR "factor analysis"[tiab] OR "factor analyses"[tiab] OR "factor structure"[tiab] OR "factor structures"[tiab] OR dimension*[tiab] OR subscale*[tiab] OR (multitrait[tiab] AND scaling[tiab] AND (analysis[tiab] OR analyses[tiab])) OR "item discriminant"[tiab] OR "interscale correlation*"[tiab] OR error[tiab] OR errors[tiab] OR "individual variability"[tiab] OR "interval variability"[tiab] OR "rate variability"[tiab] OR (variability[tiab] AND (analysis[tiab] OR values[tiab])) OR (uncertainty[tiab] AND (measurement[tiab] OR measuring[tiab])) OR "standard error of measurement"[tiab] OR sensitiv*[tiab] OR responsive*[tiab] OR (limit[tiab] AND detection[tiab]) OR "minimal detectable concentration"[tiab] OR interpretab*[tiab] OR ((minimal[tiab] OR minimally[tiab] OR clinical[tiab] OR clinically[tiab]) AND (important[tiab] OR significant[tiab] OR detectable[tiab]) AND (change[tiab] OR difference[tiab])) OR (small*[tiab] AND (real[tiab] OR detectable[tiab]) AND (change[tiab] OR difference[tiab])) OR "meaningful change"[tiab] OR "ceiling effect"[tiab] OR "floor effect"[tiab] OR "Item response model"[tiab] OR IRT[tiab] OR Rasch[tiab] OR "Differential item functioning"[tiab] OR DIF[tiab] OR "computer adaptive testing"[tiab] OR "item bank"[tiab] OR "cross-cultural equivalence"[tiab])	4
5	NOT (‘delphi-technique’[ti] OR cross-sectional[ti] OR "addresses"[Publication Type] OR "biography"[Publication Type] OR "case reports"[Publication Type] OR "comment"[Publication Type] OR "directory"[Publication Type] OR "editorial"[Publication Type] OR "festschrift"[Publication Type] OR "interview"[Publication Type] OR "lectures"[Publication Type] OR "legal cases"[Publication Type] OR "legislation"[Publication Type] OR "letter"[Publication Type] OR "news"[Publication Type] OR "newspaper article"[Publication Type] OR "patient education handout"[Publication Type] OR "popular works"[Publication Type] OR "congresses"[Publication Type] OR "consensus development conference"[Publication Type] OR "consensus development conference, nih"[Publication Type] OR "practice guideline"[Publication Type]) NOT ("animals"[MeSH Terms] NOT "humans"[MeSH Terms])	5
6		4 NOT 5
7		3 AND 6

### Data management

We will utilise our previously published data management protocol [32]. Essentially, we will keep an audit trial of searches, deduplication, and screening process on the principal investigators’ database accounts (e.g. PubMed, Mendeley, and Rayyan accounts, respectively). We will first deduplicate the search hits in Mendeley and thereafter in Rayyan. Last, we will also create a live document outlining all data collection processes (e.g. search hits per database and date of search); this will be available to the review team.

### Data collection process

The principal investigator, J. D., will collaboratively search the databases assisted by the librarian. The searches will be conducted from 1992, the year in which the Hope Index was initially published, to the searches’ penultimate date. The retrieved articles will be exported to Mendeley for first-stage deduplication. Afterwards, the articles will be exported to Rayyan for second-level deduplication and screening. Two researchers (B. S. and M. M.) will screen articles by title and abstract. The researchers will screen the articles independently of each other. Before screening, we will perform a standardisation process where both researchers will screen the same 50 articles, and the agreement rate will be calculated. The differences will be discussed as part of the training, and training will be stopped when the researchers attain at least a modified kappa ≥ 0.80. Afterwards, two researchers (T. M. and L. D.) will extract data from the full-text articles meeting all selection criteria. Disagreements will be resolved through discussions, with J. D., the most senior researcher, making the final decision in the event of an impasse.

### Outcomes and prioritisation

We will extract contextual details broadly categorised into study background characteristics, research methodology, and key results. First, we will extract data on the study characteristics such as setting, participants’ demographics (e.g. sex, age), and clinical characteristics (e.g. healthy population/specific condition(s)). We will also collect data on methodological aspects, for example administrative mode (physical/digital, self/interviewer-administered) and sampling (e.g. sample size, sampling methods, amongst other pertinent variables to evaluate the risk of bias across the studies and to contextualise the analysed studies and assess the feasibility of the HI index). Last, given the variations in psychometrics terminology/definitions, we will apply the COSMIN taxonomy to standardise the data collection process and collect data on the HI psychometrics across studies — see Table 3 [27, 33]. For this review, structural and cross-cultural validity will be the primary outcomes. Structural validity is considered the most important psychometric property, as the rest of psychometrics (e.g. reliability, responsiveness) depends on robust structural validity [27, 33]. Secondary outcomes include reliability indices (e.g. internal consistency), construct validity, responsiveness, hypothesis testing, and interpretability.

**Table 3:** Operational definitions of psychometric properties [27, 33]

Term			Definition
Domain	Measurement property	Aspect of a measurement property	
Reliability			The degree to which the measurement is free from measurement error
Reliability (extended definition)			The extent to which scores for patients who have not changed are the same for repeated measurement under several conditions, e.g. using different sets of items from the same PROM (internal consistency), over time (test‐retest), by different persons on the same occasion (inter‐ rater), or by the same persons (i.e. raters or responders) on different occasions (intra‐rater)
	Internal consistency		The degree of the interrelatedness among the items
	Reliability		The proportion of the total variance in the measurements which is due to "true’^†^ differences between patients
	Measurement error		The systematic and random error of a patient's score that is not attributed to true changes in the construct to be measured
Validity			The degree to which a PROM measures the construct(s) it purports to measure
	Content validity		The degree to which the content of a PROM is an adequate reflection of the construct to be measured
		Face validity	The degree to which (the items of) a PROM indeed looks as though they are an adequate reflection of the construct to be measured
	Construct validity		The degree to which the scores of a PROM are consistent with hypotheses *(for instance with regard to internal relationships, relationships to scores of other instruments, or differences between relevant groups)* based on the assumption that the PROM validly measures the construct to be measured
		Structural validity	The degree to which the scores of a PROM are an adequate reflection of the dimensionality of the construct to be measured
		Hypotheses testing	Item construct validity
		Cross-cultural validity	The degree to which the performance of the items on a translated or culturally adapted PROM is an adequate reflection of the performance of the items of the original version of the PROM
	Criterion validity		The degree to which the scores of a PROM are an adequate reflection of a 'gold standard'
Responsiveness			The ability of a PROM to detect change over time in the construct to be measured
	Responsiveness		Idem responsiveness
Interpretability*			Interpretability is the degree to which one can assign qualitative meaning ‐ that is, clinical or commonly understood connotations – to a PROM's quantitative scores or change in scores

^†^ The word "true" must be seen in the context of the CTT, which states that any observation is composed of two components – a true score and error associated with the observation. 'True' is the average score that would be obtained if the scale was given an infinite number of times. It refers only to the consistency of the score, and not to its accuracy. ^*^ Interpretability is not considered a measurement property, but an important characteristic of a measurement instrument

### Risk-of-bias individual studies

We will use the COSMIN risk-of-bias checklist to assess the risk of bias (RoB) across the retrieved studies [28]. The COSMIN checklist is a comprehensive tool for measuring the methodological rigour of psychometric evaluation/validation studies. Not all eligible studies will be primarily designed as validation studies. For instance, studies evaluating construct validity, for example a cross-sectional study exploring the correlation between hope and depression, are likely to be prone to reporting bias. To ensure a more fair/objective assessment, we will contact authors to provide missing information for a more truthful rating. The RoB assessments will be done by two researchers (B. S. and L. D.) independently; differences will be resolved through discussions [28].

### Psychometric properties and data extraction

In keeping with the COSMIN methodology, we will rate the quality of psychometrics per these four categories: sufficient (+), insufficient (−), or indeterminate (?) — see Table 4. Positive ratings indicate an ideal tool with desirable psychometric properties [27].

**Table 4:** Updated criteria for good measurement properties [21, 29]

Measurement property	Rating^1^	Criteria
Structural validity	+	CTT: CFA: CFI or TLI or comparable measure > 0.95 OR RMSEA < 0.06 OR SRMR < 0.08^2^ IRT/Rasch: No violation of unidimensionality^3^: CFI or TLI or comparable measure > 0.95 OR RMSEA < 0.06 OR SRMR < 0.08 *AND* No violation of local independence: residual correlations among the items after controlling for the dominant factor < 0.20 OR Q3's < 0.37 *AND* No violation of monotonicity: adequate looking graphs OR item scalability > 0.30 *AND* Adequate model fit: IRT: χ^2^ > 0.01 Rasch: Infit and outfit mean squares ≥ 0.5 and ≤ 1.5 OR Z‐ standardised values > ‐2 and < 2
	?	CTT: Not all information for " + " was reported IRT/Rasch: Model fit not reported
	–	Criteria for " + " not met
Internal consistency	+	At least low evidence^4^ for sufficient structural validity^5^ AND Cronbach's alpha(s) ≥ 0.70 for each unidimensional scale or subscale^6^
	?	Criteria for "At least low evidence^4^ for sufficient structural validity^5^" not met
	–	At least low evidence^4^ for sufficient structural validity^5^ AND Cronbach’s alpha(s) < 0.70 for each unidimensional scale or subscale^6^
Reliability	+	ICC or weighted kappa ≥ 0.70
	? –	ICC or weighted kappa not reported ICC or weighted kappa < 0.70
Measurement error	+ ? –	SDC or LoA < MIC^5^ MIC not defined SDC or LoA > MIC^5^
Hypotheses testing for construct validity	+	The result is in accordance with the hypothesis^7^
	?	No hypothesis defined (by the review team)
	–	The result is not in accordance with the hypothesis^7^
Cross‐cultural validity\measurement invariance	+	No important differences found between group factors (such as age, gender, language) in multiple group factor analysis OR no important DIF for group factors (McFadden's R^2^ < 0.02)
	?	No multiple group factor analysis OR DIF analysis performed
	–	Important differences between group factors OR DIF was found
Criterion validity	+	Correlation with gold standard ≥ 0.70 OR AUC ≥ 0.70
	?	Not all information for ' + ' reported
	–	Correlation with gold standard < 0.70 OR AUC < 0.70
Responsiveness	+	The result is in accordance with the hypothesis^7^ OR AUC ≥ 0.70
	?	No hypothesis defined (by the review team)
	–	The result is not in accordance with the hypothesis^7^ OR AUC < 0.70

Key: *AUC* area under the curve, *CFA* confirmatory factor analysis, *CFI* comparative fit index, *CTT* classical test theory, *DIF* differential item functioning, *ICC* intraclass correlation coefficient, *IRT* item response theory, *LoA* limits of agreement, *MIC* minimal important change, *RMSEA* root mean square error of approximation, *SEM* standard error of measurement, *SDC* smallest detectable change, *SRMR* standardised root mean residuals, *TLI* Tucker‐Lewis index

^1^“+” = sufficient, ” –“ = insufficient, “?” = indeterminate

^2^To rate the quality of the summary score, the factor structures should be equal across studies

^3^unidimensionality refers to a factor analysis per subscale, while structural validity refers to a factor analysis of a (multidimensional) patient‐reported outcome measure

^4^As defined by grading the evidence according to the GRADE approach

^5^This evidence may come from different studies

^6^The criteria ‘Cronbach alpha

^7^The results of all studies should be taken together and it should then be decided if 75% of the results are inaccordance with the hypotheses

### Best evidence synthesis

To appraise the robustness of the psychometric performance of the HI, we will synthesize the evidence across studies measuring the same psychometric property (e.g. structural validity). We will use the Cochrane Collaboration Back Review Group updated method guidelines for systematic reviews [34] as outlined in Table 5 to collate the evidence per psychometric property. The GRADE guidelines consider the RoB and quality of psychometric property to classify the overall evidence into these overall ratings: very low, low, moderate, and high.

**Table 5:** GRADE checklist- best evidence synthesis [30]

Quality level	Definition/criterion
High	We are very confident that the true measurement property lies close to that of the estimate of the measurement property
Moderate	We are moderately confident in the measurement property estimate: the true measurement property is likely to be close to the estimate of the measurement property, but there is a possibility that it is substantially different
Low	Our confidence in the measurement property estimate is limited: the true measurement property may be substantially different from the estimate of the measurement property
Very low	We have very little confidence in the measurement property estimate: the true measurement property is likely to be substantially different from the estimate of the measurement property

### Meta-analysis of psychometric properties

A meta-analysis of the psychometric properties is possible as the review focuses on the psychometric performance of the HI. However, we expect heterogeneity in the methodologies across studies (e.g. study design, sample size); the random effects model will be used for metanalysis [35]. These psychometric properties are amenable to meta-analysis: internal consistency, as measured using Cronbach’s alpha (α), reliability per intraclass correlation coefficient (ICC)), and hypothesis testing/construct validity evaluated using co-relation coefficients [28]. For instance, we will first enter the α and associated confidence intervals. The inverse-variance method will be used; study weights will be assigned to account for heterogeneity across studies [35]. We will use the mean difference approach for continuous outcomes (e.g. α, ICC). Studies with small standard deviations will be assigned greater weights as they depict more reliability/consistency. The mean difference is appropriate for the current study as we assume that differences in psychometric performance are a function of differences in populations. Results will be visualised using forests plots; we will visualise individual studies, pooled effect estimates, and the associated 95% confidence. We also visually inspect forest plots to assess heterogeneity. Also, heterogeneity will be assessed using the I2 statistic and interpreted: low (0–40%), moderate (30–60%), substantial (50–90%), and considerable (75–100%) heterogeneity. Additionally, the Cochrane Q test (chi-square statistic) and associated *p*-values will test the null hypothesis; there is no heterogeneity across the studies [35]. A conservative *p*-value of *p* = 0.10 will be used to cater for the inclusion of small sample studies. We will also perform subgroup analysis per RoB classifications according to the COSMIN classifications [28]. In the presence of high heterogeneity, we will perform a sensitivity analysis after performing a stepwise omission of studies with high heterogeneity to test the impact on the pooled effect. We anticipate that some of the outcome data will be non-normally distributed, violating the meta-analytic technique, hence the need for a sensitivity analysis to assess the robustness of the pooled effect size. We will also perform a sensitivity analysis to evaluate the effect of changing the meta-analytic model (e.g. the fixed-effect model). Publication bias will be assessed through the Egger’s linear regression test and by visual inspection of the funnel plots. Also, we will perform a meta-regression of the residuals and plot the Galbraith graph to increase the robustness of the publication bias assessment given the potential limitation of funnel plots as they do not directly accommodate effect heterogeneity [36]. Meta-analysis will be performed per Cochrane guidelines [35] using Cochrane’s RevMan software and Stata (Version 17.0).

## Discussion

The proposed systematic review and meta-analysis will appraise the collective evidence of the psychometric robustness of HI. The proposed review builds upon past qualitative reviews which have yielded inconclusive evidence of the psychometric properties of the HI, yet it is a widely applied hope outcome measure. Given the propensity towards positive psychological interventions, including hope interventions, there is a need for using reliable and valid outcome measures with known measurement/psychometric properties. The review will also assist in identifying psychometrics that may require further evaluation, including recommendations on the utility of the HI.

## Supplementary Information

Supplementary Material 1.

## Data Availability

Not applicable.

## Abbreviations

CINAHL: Cumulative Index of Nursing and Allied Health Literature
COSMIN: COnsensus-based Standards for the selection of health Measurement Instruments
GRADE: Grading of Recommendations Assessment, Development, and Evaluation
HRQoL: Health-related quality of life
LMICS: Low- and middle-income countries
PRISMA-P: Preferred Reporting Items of Systematic reviews and Meta-Analyses Protocol
