# Supplementary material for: An evaluation of psychometric properties and utility of the Hope Index (HI): a systematic review and meta-analysis protocol
Source: Syst Rev. 2026 Mar 3;15:127. doi: 10.1186/s13643-026-03128-7 (PMC13069760; doi:10.1186/s13643-026-03128-7)
Supplement: Supplementary file 1 [file 13643_2026_3128_MOESM1_ESM.odt]

PRISMA-P 2015 Checklist	

# This checklist has been adapted for use with protocol submissions to Systematic Reviews from Table 3 in Moher D et al: Preferred reporting items for systematic review and meta-analysis protocols (PRISMA-P) 2015 statement. Systematic Reviews 2015 4:1

| Section/topic | # | Checklist item | Information reported | Page number(s) |
| --- | --- | --- | --- | --- |
| Yes | No |
| ADMINISTRATIVE INFORMATION |
| Title |
| Identification | 1a | Identify the report as a protocol of a systematic review |  |  | 1 |
| Update | 1b | If the protocol is for an update of a previous systematic review, identify as such |  |  | N/A |
| Registration | 2 | If registered, provide the name of the registry (e.g., PROSPERO) and registration number in the Abstract |  |  | 2 |
| Authors |
| Contact | 3a | Provide name, institutional affiliation, and e-mail address of all protocol authors; provide physical mailing address of corresponding author |  |  | 1 |
| Contributions | 3b | Describe contributions of protocol authors and identify the guarantor of the review |  |  | 11 |
| Amendments | 4 | If the protocol represents an amendment of a previously completed or published protocol, identify as such and list changes; otherwise, state plan for documenting important protocol amendments |  |  | N/A |
| Support |
| Sources | 5a | Indicate sources of financial or other support for the review |  |  | 11 |
| Sponsor | 5b | Provide name for the review funder and/or sponsor |  |  | 11 |
| Role of sponsor/funder | 5c | Describe roles of funder(s), sponsor(s), and/or institution(s), if any, in developing the protocol |  |  | 11 |
| INTRODUCTION |
| Rationale | 6 | Describe the rationale for the review in the context of what is already known |  |  | 3 - 4 |
| Objectives | 7 | Provide an explicit statement of the question(s) the review will address with reference to participants, interventions, comparators, and outcomes (PICO) |  |  | 4 |
| METHODS |
| Eligibility criteria | 8 | Specify the study characteristics (e.g., PICO, study design, setting, time frame) and report characteristics (e.g., years considered, language, publication status) to be used as criteria for eligibility for the review |  |  | 4 - 5 |
| Information sources | 9 | Describe all intended information sources (e.g., electronic databases, contact with study authors, trial registers, or other grey literature sources) with planned dates of coverage |  |  | 5 |
| Search strategy | 10 | Present draft of search strategy to be used for at least one electronic database, including planned limits, such that it could be repeated |  |  | 5 – 6 |
| STUDY RECORDS |
| Data management | 11a | Describe the mechanism(s) that will be used to manage records and data throughout the review |  |  | 6 |
| Selection process | 11b | State the process that will be used for selecting studies (e.g., two independent reviewers) through each phase of the review (i.e., screening, eligibility, and inclusion in meta-analysis) |  |  | 6 – 7 |
| Data collection process | 11c | Describe planned method of extracting data from reports (e.g., piloting forms, done independently, in duplicate), any processes for obtaining and confirming data from investigators |  |  | 6 - 7 |
| Data items | 12 | List and define all variables for which data will be sought (e.g., PICO items, funding sources), any pre-planned data assumptions and simplifications |  |  | 6 - 7 |
| Outcomes and prioritization | 13 | List and define all outcomes for which data will be sought, including prioritization of main and additional outcomes, with rationale |  |  | 6 - 7 |
| Risk of bias in individual studies | 14 | Describe anticipated methods for assessing risk of bias of individual studies, including whether this will be done at the outcome or study level, or both; state how this information will be used in data synthesis |  |  | 8 |
| DATA |
| Synthesis | 15a | Describe criteria under which study data will be quantitatively synthesized |  |  | 8 - 11 |
| 15b | If data are appropriate for quantitative synthesis, describe planned summary measures, methods of handling data, and methods of combining data from studies, including any planned exploration of consistency (e.g., I 2, Kendall’s tau) |  |  | 10 - 11 |
| 15c | Describe any proposed additional analyses (e.g., sensitivity or subgroup analyses, meta-regression) |  |  | N/A |
| 15d | If quantitative synthesis is not appropriate, describe the type of summary planned |  |  | N/A |
| Meta-bias(es) | 16 | Specify any planned assessment of meta-bias(es) (e.g., publication bias across studies, selective reporting within studies) |  |  | N/A |
| Confidence in cumulative evidence | 17 | Describe how the strength of the body of evidence will be assessed (e.g., GRADE) |  |  | 10 |
